# Upregulation of Cleavage and Polyadenylation Specific Factor 4 in Lung Adenocarcinoma and Its Critical Role for Cancer Cell Survival and Proliferation

**DOI:** 10.1371/journal.pone.0082728

**Published:** 2013-12-16

**Authors:** Wangbing Chen, Wei Guo, Mei Li, Dingbo Shi, Yun Tian, Zhenlin Li, Jingshu Wang, Lingyi Fu, Xiangsheng Xiao, Quentin Qiang Liu, Shusen Wang, Wenlin Huang, Wuguo Deng

**Affiliations:** 1 State Key Laboratory of Oncology in South China, Collaborative Innovation Center of Cancer Medicine, Sun Yat-sen University Cancer Center, Guangzhou, China; 2 Institute of Cancer Stem Cell, Dalian Medical University Cancer Center, Dalian, China; 3 State Key Laboratory of Targeted Drug for Tumors of Guangdong Province, Guangzhou Double Bioproduct Inc., Guangzhou, China; Georgia Regents University, United States of America

## Abstract

Cleavage and polyadenylation specific factor 4 (CPSF4), a member of CPSF complex, plays a key role in mRNA polyadenylation and mRNA 3′ ends maturation. However, its possible role in lung cancer pathogenesis is unknown. In this study, we investigated the biological role and clinical significance of CPSF4 in lung cancer growth and survival and elucidated its underlying molecular mechanisms. We found that CPSF4 was highly expressed in lung adenocarcinoma cell lines and tumor tissue but was undetectable in 8 normal human tissues. We also found that CPSF4 overexpression was correlated with poor overall survival in patients with lung adenocarcinomas (*P*<0.001). Multivariate survival analyses revealed that higher CPSF4 expression was an independent prognostic factor for overall survival of the patients with lung adenocarcinomas. Suppression of CPSF4 by siRNA inhibited lung cancer cells proliferation, colony formation, and induced apoptosis. Mechanism studies revealed that these effects were achieved through simultaneous modulation of multiple signaling pathways. Knockdown of CPSF4 expression by siRNA markedly inhibited the phosphorylation of PI3K, AKT and ERK1/2 and JNK proteins. In contrast, the ectopic expression of CPSF4 had the opposite effects. Moreover, CPSF4 knockdown also induced the cleavage of caspase-3 and caspse-9 proteins. Collectively, these results demonstrate that CPSF4 plays a critical role in regulating lung cancer cell proliferation and survival and may be a potential prognostic biomarker and therapeutic target for lung adenocarcinoma.

## Introduction

Primary lung cancer is the leading cause of cancer-related deaths worldwide [1]. Non-small cell lung cancer (NSCLC) is a major form of lung cancer and its incidence has been increasing in the past several decades. Novel insight into the biology of NSCLC have improved outcome for certain patients [2], [3], however, the 5-year overall survival rate for patients with NSCLC has not been markedly improved [4]. Therefore, there is an urgent need for further understanding the molecular mechanisms in lung cancer tumorigenesis and for identifying new therapeutic targets to improve the prognosis of patients.

Cleavage and Polyadenylation Specific Factor 4 (CPSF4; alternatively known as CPSF30 or NEB1) was originally discovered as an essential component of the 3' end processing machinery of cellular pre-mRNAs. The protein is a member of CPSF complex, which was reported to participate mRNA 3′ ends maturation and play a key role in polyadenylation of mRNA with other members of CPSF complex[5]–[9]. It has been proposed that these mRNA 3′ ends maturation factors including CPSF4 maybe link to tumor suppression [10], [11]. In contrast, other studies identify that a potential oncogenic role of these mRNA 3′ ends processing factors in multiple human cancers [12]–[15]. In influenza virus-infected cells, CPSF4 protein interacted with virus NS1 protein and inhibited 3' end cleavage and polyadenylation of host pre-mRNAs [16]. CPSF4 protein functionally interacted with tumor suppressor Cdc73 and regulated the transcription of specific gene INTS6 in HeLa cell [10]. Although the function of CPSF4 has been examined in different model systems, its potential role in tumors has not been fully investigated due to a lack of data regarding CPSF4 expression and tumor cell growth.

In this study, we investigated the expression and clinical significance of CPSF4 in lung adenocarcinoma and explored its roles and underlying mechanisms for cancer cell survival and proliferation. We have shown that CPSF4 is overexpressed in a subset of lung adenocarcinomas, which correlates with poor overall survival. Knockdown of CPSF4 in lung cancers leads to reduced cell growth, proliferation and increased apoptosis in lung adenocarcinoma cell lines with high endogenous levels of CPSF4. Our results indicate that CPSF4 may be a potential prognostic biomarker and therapeutic target for lung adenocarcinoma.

## Results

### CPSF4 is highly expressed in lung adenocarcinomas cell lines and tumor tissues

**Figure 1:**
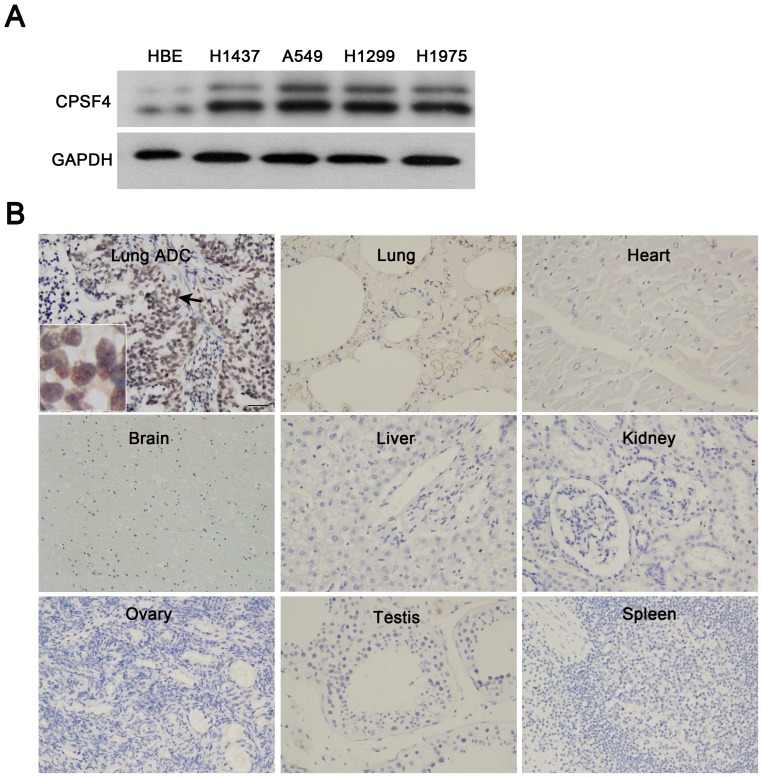
CPSF4 is highly expressed in lung adenocarcinoma cell lines and tumor tissues. (**A**) The expression of CPSF4 in human normal lung cell HBE and various lung adenocarcinoma cell lines was analyzed by Western blot. (**B**) The expression of CPSF4 in 8 normal human tissues and lung adenocarcinoma tissues was detected by immunohistochemical staining. (magnification, ×200). ADC, adenocarcinoma.

We first examined the expression of CPSF4 protein in lung adenocarcinomas cell lines by Western blotting. The lung adenocarcinomas cell lines expressed the high levels of CPSF4 proteins as compared with the HBE (Fig. 1A). We also detected the expression of CPSF4 protein in lung adenocarcinomas tissue and 8 normal human tissues (heart, lung, liver, kidney, brain, spleen, ovary, and testis) by immunohistochemistry assay. As shown in Figure 1B, a positive staining of CPSF4 was observed in the nucleus of lung cancer cells, whereas CPSF4 staining could not be detected in all 8 normal tissues, suggesting that CPSF4 might be a potential biomarker for lung adenocarcinomas.

### CPSF4 overexpression is associated with poor prognosis of lung adenocarcinomas patients

**Figure 2:**
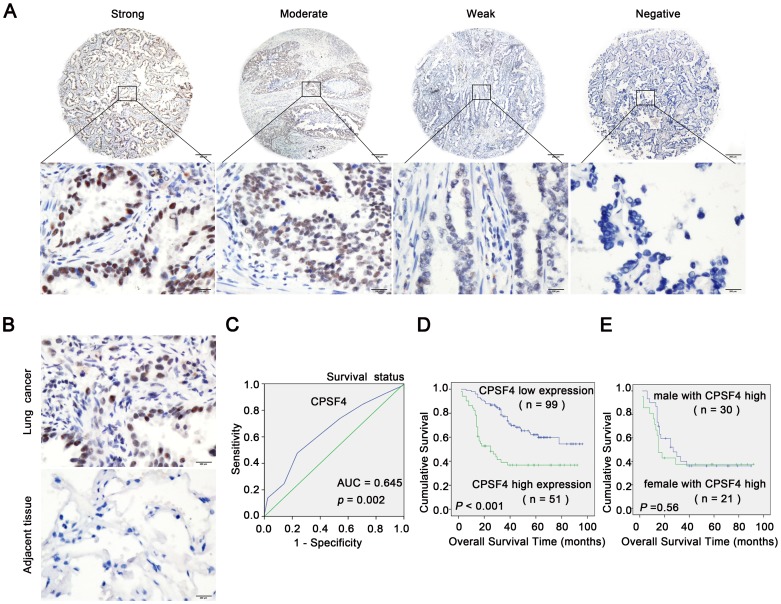
High CPSF4 expression correlates with shorter overall survival in patients with lung adenocarcinoma. (**A**) Representative examples for strong, moderate, weak and negative CPSF4 expression in lung adenocarcinoma tissues. Upper panels, ×40; lower panels, ×400, from the area of the box in upper panel respectively. (**B**) Comparison of CPSF4 expression levels between lung adenocarcinoma tissues and adjacent non-malignant lung tissues by immunohistochemistry in the same patient. Representative images of paired lung adenocarcinoma tissues and adjacent non-malignant lung tissues were shown (magnification, ×400).(**C**) ROC curve analysis was used to determine the cut-off score for high expression of CPSF4 protein in lung adenocarcinoma tissues. The sensitivity and specificity for OS were plotted; *p* = 0.002. (**D**) Kaplan–Meier analysis of overall survival with high or low CPSF4 expression (*p*<0.001, log-rank test). (**E**) Kaplan–Meier analysis of overall survival of male and female patients with high CPSF4 expression (*p* = 0.56).

To investigate the clinicopathologic significance of CPSF4 in lung adenocarcinomas, we carried out immunohistochemical staining on a tissue microarray. CPSF4-positive staining was observed in the nucleus of lung cancer cells, but staining was negative in adjacent non-malignant lung tissues (Fig. 2A and 2B).

To develop a reasonable cut-off score of CPSF4 for further survival analysis, we subjected each IHC score to ROC curve analysis with respect to patient outcome. As shown in (Fig. 2C), the CPSF4 IHC cut-off scores for OS was 5.0. Thus, the expression of CPSF4 in each sample was subsequently classified as either high level (score > 5) or low level (score ≤ 5). The clinicopathological characteristics of the 150 patients with lung adenocarcinomas are shown in Table 1A. We found that the high CPSF4 expression rate was higher in patients with later N classification (P = 0.033, Chi-Square test) (Table 1). There was no significant correlation between CPSF4 expression and other clinicopathologic parameters, such as patient sex, age (≥60 vs. <60 years), T classification (P>0.05, Table 1). Survival analyses showed that high expression of CPSF4 significantly correlated with shorter overall survival time in patients with lung adenocarcinomas (P<0.001, log-rank test; Fig. 2D). Survival analyses also showed that there was no significant difference of the overall survival time between male and female patient with CPSF4 high levels (P = 0.56, log-rank test; Fig. 2E).

**Table 1:** Association of CPSF4 expression with patient’s clinicopathological features in human lung adenocarcinoma.

	Total (*n* = 150)	CPSF4 low expression (*n* = 99)	CPSF4 high expression (*n* = 51)	*p*
Gender				
Male	80	50(62.5%)	30(37.5%)	0.333
Female	70	49(70.0%)	21(30.0%)	
Age,y				
≥60	80	50(62.5%)	30(37.5%)	0.333
<60	70	49(70.0%)	21(30.0%)	
pT factor				
T1+T2	118	80(67.8%)	38(32.2%)	0.372
T3+T4	32	19(59.4%)	13(40.6%)	
pN factor				
N0+N1	77	57(74.0%)	20(26.0%)	0.033^a^
N2+N3	73	42(57.5%)	31(42.5%)	

^a^ *P*<0.05. Abbreviations: ADC, adenocarcinoma;

We also used a univariate analysis to evaluate associations between patient prognosis and several clinicopathologic factors including CPSF4 expression (high vs. low), gender (male vs. female), age (≥60 vs. <60 years), pT stage (tumor size; T3-T4 vs. T1-T2) and pN stage (lymph node metastasis; N2-N3 vs. N0–N1). All of these parameters except gender and age were significantly associated with inferior prognosis (Table 2). Multivariate analysis further indicated that high CPSF4 level and pN stage were independent prognostic factors for overall survival of patients with lung adenocarcinomas (Table 2). All of these findings suggest that CPSF4 may have an important role in promoting more aggressive behavior of lung cancer cells.

**Table 2:** Cox proportional hazards model analysis of prognostic factors in patients with lung adenocarcinoma.

	HR	95% CI	Unfavorable/Favorable	*p*
Univariate analysis				
CPSF4	2.706	1.657–4.418	High/low	<0.0001^a^
Gender	1.243	0.764–2.023	Male/female	0.381
Age,y	1.199	0.733–1.960	≥60/<60	0.470
pT factor	2.568	1.516–4.349	T3+T4/T1+T2	0.001^a^
pN factor	3.993	2.330–6.845	N2+N3/N0+N1	<0.0001^a^
Multivariate analysis				
CPSF4	2.692	1.642–4.416	High/low	<0.0001^a^
pT factor	1.537	0.888–2.659	T3+T4/T1+T2	0.124
pN factor	3.496	1.981–6.168	N2+N3/N0+N1	<0.0001^a^

^a^ *P*<0.05. Abbreviations: ADC, adenocarcinoma; HR, hazard ratio; CI, confidence interval;

### CPSF4 knockdown inhibits lung adenocarcinoma cell proliferation and survival

**Figure 3:**
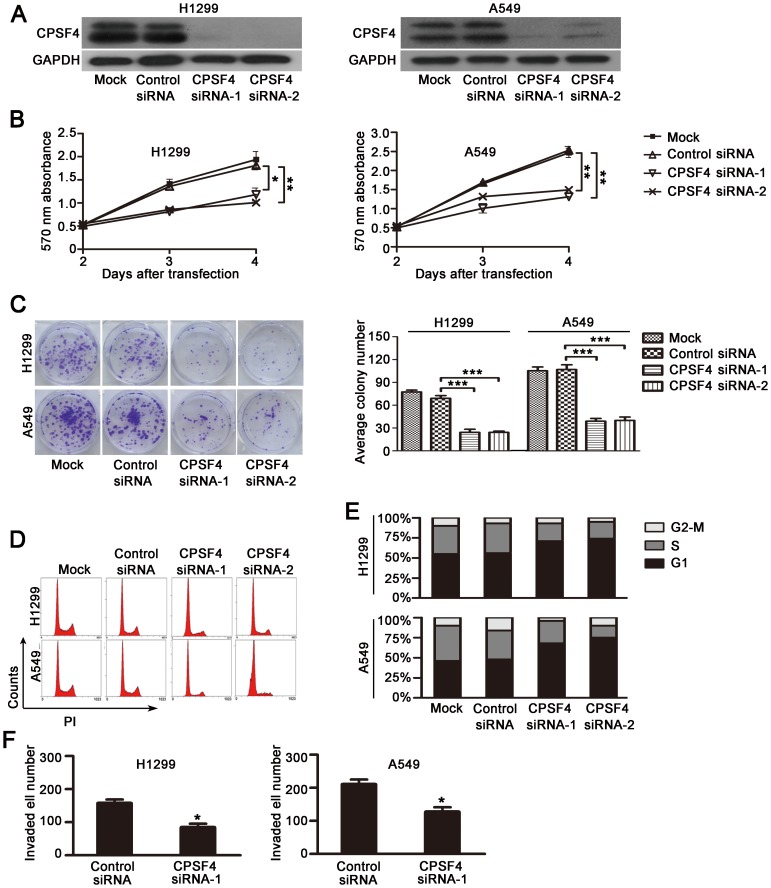
Knockdown of CPSF4 inhibits proliferation and survival of lung adenocarcinoma cell lines H1299 and A549. (**A**) Analysis of CPSF4 expression by Western blot 3 days after siRNA transfection. Mock, vehicle control; Control siRNA, nonspecific siRNA; CPSF4 siRNA-1 and CPSF4 siRNA-2, CPSF4 specific siRNA. (**B**) Cell viability was measured by MTT assay. The mean and SD obtained from three independent experiments are plotted (*, *P*<0.05,**, *P*<0.01). (**C**) Cells were treated with CPSF4 siRNA or control siRNA (50 nmol/L) every 3 days and grown for 14 days; colonies stained with crystal violet were counted. Results are shown as the mean ± SD of three independent experiments (***, *P*<0.001). (**D, E**) At 3 days after siRNA transfection, cell cycle was analyzed by flow cytometry using PI staining. Representative data from three independent experiments are shown (**D**). Percentage of cells at each phase (**E**). (**F**) H1299 and A549 cells were seeded in the chamber of transwells coated with Matrigel matrix. The invasiveness of cells was determined by counted cells passing through Matrigel matrix to the basal side of transwells. *P<0.05 when compared with the nonspecific control siRNA group.

To assess whether the cells expressing high levels of CPSF4 rely on it for survival and proliferation, we transfected siRNA oligonucleotides targeting CPSF4 into H1299 and A549 cells, which express significant levels of CPSF4. The results showed that the DC-based CPSF4 siRNA nanoparticles markedly downregulated CPSF4 protein expression (Fig. 3A), and the knockdown of CPSF4 significantly inhibited cell viability as compared with the transfection with the nonspecific siRNA groups by MTT assay (Fig. 3B).

To confirm the CPSF4 siRNA-mediated inhibition of cell growth, we next performed colony formation experiments and found that CPSF4 knockdown significantly decreased colony formation in H1299 and A549 cells (Fig. 3C). Since a growth inhibitory effect was observed in siCPSF4-transfected cells, we analyzed the transfectants for cell-cycle parameters by flow cytometry. As shown in Fig. 3D and 3E, the accumulation of G1 cells markedly increased in the CPSF4 siRNA–treated group as compared with the nonspecific siRNA controls (75% vs. 55% in H1299 and 73% vs. 46% in A549). In addition, we also showed that knockdown of CPSF4 by siRNA considerably inhibited cell invasion in both H1299 and A549 cells (Fig. 3F). These results indicate that knockdown of CPSF4 in lung cancer cells resulted in a significant inhibitions in cell proliferation and cell invasion.

### CPSF4 knockdown inactivates PI3K/AKT and MAPK signaling

**Figure 4:**
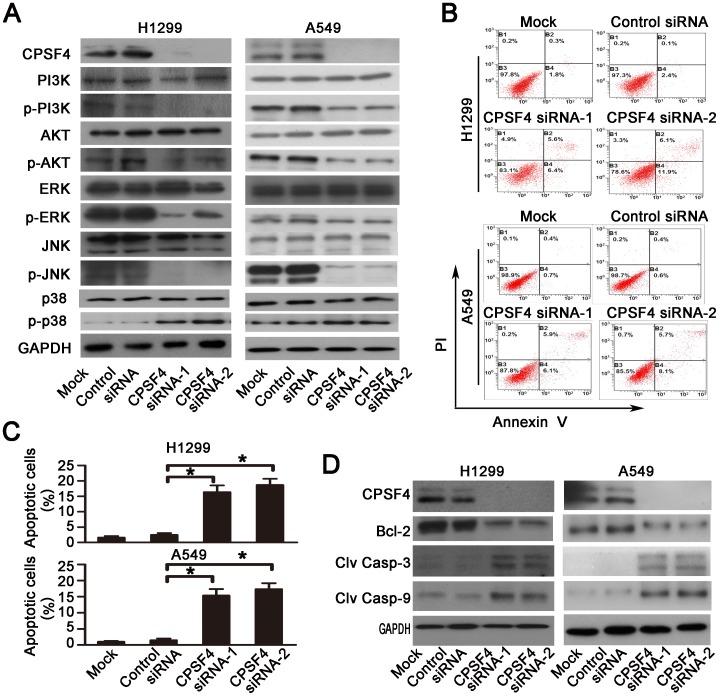
Knockdown of CPSF4 inhibits PI3K/AKT, MAPK signaling and activates caspase-dependent apoptotic pathway in H1299 and A549 cells. (**A**) At 72 hours after siRNA treatment, the expression of CPSF4 protein and the total and phosphorylated Akt, PI3K, ERK1/2, JNK and p38 proteins in H1299 and A549 was detected by Western blot. GAPDH served as the loading control. (**B**) Apoptosis in H1299 and A549 was determined by flow cytometry 72 h after siRNA transfection using an Annexin V-FITC/PI-staining kit. The representative data from three independent experiments are shown. (**C**) Apoptosis was calculated in terms of the FITC-positive in cells. Results are shown as the mean ± SD of three independent experiments (*, *P*<0.05). (**D**) At 72 hours after siRNA treatment, the expression of Bcl-2, cleaved caspase-3 or 9 proteins in H1299 and A549 was detected by Western blot. GAPDH was used as the loading control.

To identify the potential molecular mechanisms by which CPSF4 knockdown inhibited lung cancer cell survival and proliferation, we analyzed the activities of several pro-survival proteins by Western blot analysis. The results showed that CPSF4 knockdown in both H1299 and A549 cells dramatically suppressed the phosphorylation of PI3K, AKT, ERK1/2 and JNK proteins, but considerably increased p38 protein phosphorylation (Fig. 4A), thereby leading to an inactivation of the PI3K/AKT and MAPK signaling pathways.

### CPSF4 knockdown induces apoptosis in lung cancer cells

We next investigated the effect of CPSF4 knockdown on apoptosis by Annexin V-FITC/PI staining-based FACS analysis (Fig. 4B). Knockdown of CPSF4 by siRNA resulted in a significant induction of cell apoptosis in both H1299 and A549 cells (Fig. 4C). Activation of caspases is an important event in apoptosis signaling pathway. We next detected the effect of CPSF4 knockdown on the expression and activation of two key pro-apoptotic proteins: caspase-3 and caspase-9, in H1299 and A549 cells by Western blot. As shown in Fig. 4D, CPSF4 knockdown induced the activation of caspase-3 and -9, resulting in a marked increase in the levels of cleaved caspase-3 and cleaved caspase-9 proteins. Knockdown of CPSF4 also downregulated the expression of Bcl-2, an important anti-apoptotic protein, in both H1299 and A549 cells (Fig. 4D).Thus, these results indicated that CPSF4 might control several aspects of apoptosis signaling.

### CPSF4 overexpression promotes lung cancer cell growth and activates PI3K/AKT and MAPK signaling

**Figure 5:**
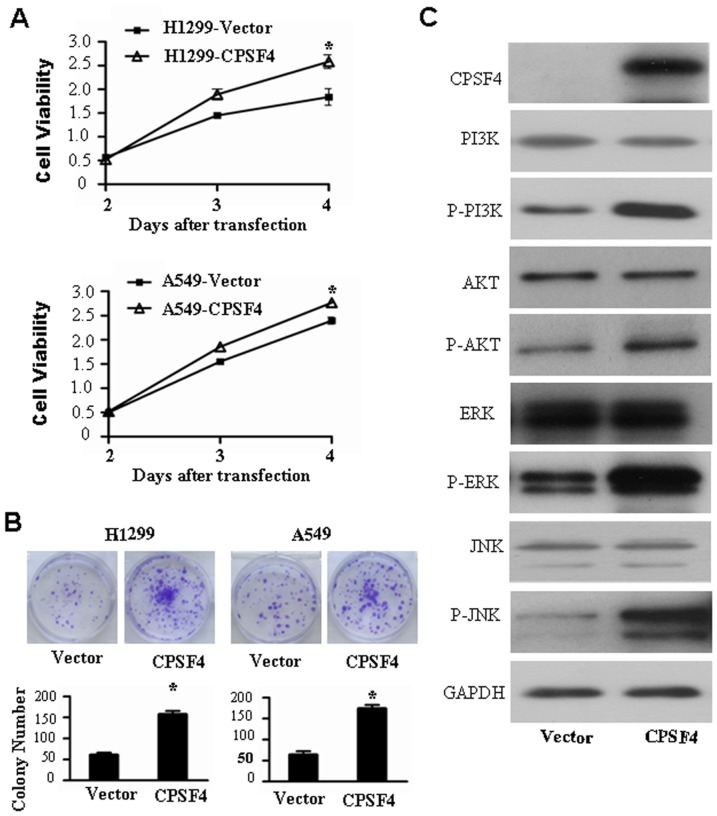
Overexpression of CPSF4 promotes cell growth and activates PI3K/AKT and MAPK signaling pathways in H1299 cells. (**A**) H1299 cells were transfected with CPSF expressing vector, at 3 or 4 days cell viability was measured by MTT assay. The data are shown as the mean ± SD of three independent experiments (*, *P*<0.05). (**B**) H1299 cells were transfected with CPSF4 expressing vectors or control vector every 3 days and grown for 8 days, the colonies were stained with crystal violet and counted. The data are shown as the mean ± SD of three independent experiments (*, *P*<0.05). (**C**) H1299 cells were transfected with CPSF expressing vector, at 72 hours after transfection, the expression of CPSF4 as well as total and phosphorylated Akt, PI3K, ERK1/2 and JNK proteins was detected by Western blot. GAPDH was used as the loading control.

To further confirm the role of CPSF4 in regulating lung cancer cell growth via the PI3K/AKT and MAPK signaling pathways, H1299 cells were transfected with an expression vector encoding CPSF4 (pcDNA3.1-CPSF4) or a control vector lacking CPSF4 (pcDNA3.1). The results showed that the CPSF4 overexpression markedly promoted cell viability (Fig. 5A) and colony formation (Fig. 5B) as compared with the transfection with the control vector groups. To identify the underlying molecular mechanisms by which CPSF4 ovexexpression increased lung cancer cell growth, we analyzed the effect of CPSF4 on the activation of AKT and MAPK signaling by Western blot. As shown in Fig. 5C, CPSF4 ovexexpression markedly upregulated the phosphorylation of PI3K, AKT, ERK1/2 and JNK proteins. These results therefore showed that the ectopic expression of CPSF4 promoted cell growth partially through the activation of the PI3K/AKT and MAPK signaling in lung cancer cells.

## Discussion

In this study, we showed that CPSF4 was highly expressed in lung adenocarcinomas cell lines and tumor tissues by comparison with normal lung cell and noncancerous lung tissues, respectively. The expression of CPSF4 in 8 normal human tissues was deficient. Moreover, clinicopathologic data from our tissue microarray showed that lung adenocarcinomas patients with highly expressed CPSF4 have shorter survival periods than those with CPSF4 low expression. Multivariate analysis showed that CPSF4 high expression is an independent prognostic factor for overall survival of lung adenocarcinomas patients. The results from our *in vitro* experiments suggested that CPSF4 exerted its oncogenic function by regulating the proliferation and apoptosis of lung adenocarcinoma cells.

CPSF4 belong the cleavage and polyadenylation specificity factor (CPSF) complex, whose other members are CPSF160, CPSF100, CPSF73 and Fip1 [17]. Recently, some studies have focused on the role of some mRNA 3′ end-processing factors in cancer, including FIP1L1, CSTF50, CSTF2 and Neo-PAP [11]–[15]. For example, Aragaki and colleagues found that the CSTF2 was highly expressed in lung cancer, whereas its expression was scarcely detectable in any of 29 normal human tissues except testis. Furthermore, the knockdown of CSTF2 by siRNA inhibited the growth of lung cancer cells. More importantly, CSTF2 overexpression was associated with poor prognosis for lung cancer patients. In this report, we provide clinical evidence that CPSF4 overexpression predicts poor prognosis in lung adenocarcinoma patients. The suppression of CPSF4 expression inhibited the growth of lung cancer cells *in vitro.* The significant prognostic value of CPSF4 could be explained by its function of pro-survival in lung cancer cells. It is still unknown why CPSF4 was overexpressed in lung cancer cells; however, based on the findings in the present study, we believe that CPSF4 may be a potential diagnostic and/or therapeutic target in lung adenocarcinomas.

In this study, we observed that siRNA-mediated CPSF4 knockdown inhibited cell growth and induced apoptosis in lung cancer cells expressing high levels of CPSF4. To investigate the underline molecular mechanisms, we examined PI3K/AKT, MAPK and apoptosis signaling pathways alteration. Inactivation of PI3K/AKT, MAPK signaling pathways by CPSF4 knockdown, as indicated by suppressed the phosphorylation of PI3K, AKT, ERK1/2 and JNK, was observed in lung cancer cell lines. The PI3K/AKT and MAPK pathways are involved in a wide variety of cellular processes such as growth, proliferation, differentiation, transcription regulation, and development [18], [19]. These two signaling pathways are activated in lung cancer and have been identified as novel target for therapy [20]–[22]. Thus, CPSF4 might exert its growth-regulating effect, at least in part, by modulating the PI3K/AKT and MAPK signaling pathways in lung cancer cells. Although further detailed analyses are necessary to determine the direct targets of CPSF4, the findings in this study imply the biological importance of CPSF4 in regulating lung cancer cell growth and survival. Thus, our results provide a rationale for pharmacologic investigation of CPSF4 as a potential novel therapeutic target in lung cancer.

In summary, CPSF4 was highly expressed in lung cancer cell lines and tumor tissues and positively correlated with poor prognosis of patients with lung adenocarcinomas. Knockdown of CPSF4 expression by siRNA significantly inhibited cell growth and induced apoptosis in lung adenocarcinoma cell lines through simultaneous inactivation of the PI3K/AKT and MAPK signaling and activation of the caspase-dependent apoptotic pathways. In contrast, the ectopic expression of CPSF4 had the opposite effects. These results therefore indicate that CPSF4 plays an important role in the regulation of growth and survival of lung adenocarcinoma cells and may be a potential therapeutic target for lung cancer.

## Materials and Methods

### Ethics statement

The study was approved by the Ethics Committee of Sun Yatsen University Cancer Center. All samples used in this study were anonymous and collected from patients for routine pathology use. No informed consent (written or verbal) was obtained for use of retrospective tissue samples from the patients in this study, since most of the patients were deceased and informed consent was not deemed necessary and waived by the Ethics Committee.

### Cell lines and cell culture

Human NSCLC cell lines (H1299, A549, H1975, H1437) were obtained from the American Type Culture Collection (ATCC, Manassas, VA) and cultured in RPMI-1640 medium (Invitrogen, Carlsbad, CA), supplemented with 10% fetal bovine serum. Normal human bronchial epithelial (HBE) was maintained in Dulbecco’s modified Eagle’s medium supplemented with 10% fetal bovine serum. Cells were maintained in a humidified atmosphere and 5% CO_2_ at 37°C.

### Western blot analysis

Cell lysates were separated by electrophoresis in 8–12% sodium dodecyl sulphate-polyacrylamide gradient minigel (SDS-PAGE) (Bio-Rad, Hercules, CA) and electrophoretically transferred to a nitrocellulose membrane (Amersham Pharmacia Biotech, Piscataway, NJ). Western blots were probed with antibodies against CPSF4 (Proteintech Group, Inc., Chicago, USA), phospho-PI3K p85 (Tyr458)/p55 (Tyr199), PI3K, phosphor-Akt (Ser473), Akt, pTyr202/Y204-ERK1/2, ERK1/2, pThr183/Tyr185-SAPK/JNK, SAPK/JNK, cleaved caspase-3, cleaved caspase-9 and GAPDH (Cell Signaling Technology, Beverly, MA).The protein bands were detected by enhanced chemiluminescence (Amersham Pharmacia Biotech, Piscataway, NJ).

### Lung adenocarcinomas tissue microarray

Tissue microarray (diameter, 1.5 mm; depth, 4 µm) used for immunostaining analysis of CPSF4 protein expression was purchased from Shanghai Outdo Biotech (Shanghai, China, http://www.superchip.com.cn/). The microarray consisted of 150 formalin-fixed, paraffin-embedded lung adenocarcinomas and corresponding adjacent non- malignant lung tissues. These tissue samples had been obtained from patients having no anticancer treatment before tumor resection. The tissue specimens were histologically examined and classified using the 2004 World Health Organization classification system [23]. Detailed clinical and pathologic information, including clinical and pathologic tumor-node-metastasis (TNM) stage and overall survival (OS) duration was available for all cases. The pathological TNM status of all lung adenocarcinomas was assessed according to the criteria of the seven edition of the American Joint Committee on Cancer (2010).

### Immunohistochemistry (IHC) and histological evaluation

Immunohistochemistry was conducted using Envisionþ Kit/HRP (DakoCytomation). In brief, slides were immersed in Target Retrieval Solution (pH 9; DakoCytomation) and boiled at 108°C for 15 minutes in an autoclave for antigen retrieval. CPSF4 antibody (1∶50 dilution) was added to each slide after blocking of endogenous peroxidase and proteins, and the sections were incubated with HRP-labeled anti-rabbit IgG [Histofine Simple Stain MAX PO (G); Nichirei] as the secondary antibody. Substrate-chromogenwas added, and the specimens were counterstained with hematoxylin. A negative control was obtained by replacing the primary antibody with a normal rabbit IgG.

The immunoreactions were evaluated independently by two pathologists blinded to the clinicopathologic information. Nuclear staining intensity was categorized: absent staining as 0, weak as 1, moderate as 2, and strong as 3. The percentage of stained cells was accessed by using a five-scale scoring system: 0 (no positive cells), 1 (<25% positive cells), 2 (25%–50% positive cells), 3 (50%–75% positive cells), and 4 (>75% positive cells). The score for each tissue was calculated by multiplying the intensity and the percentage value (the range of this calculation was therefore 0–12). The receiver operating characteristic (ROC) curve analysis was employed to determine cutoff score for tumor “high expression” by using the 0, 1-criterion. Briefly, the sensitivity and specificity for the patient outcome being studied at each score was plotted to generate a ROC curve. The score was selected as the cutoff value, which was closest to the point with both maximum sensitivity and specificity. Tumors designated as “low expression” for CPSF4 was those with scores below or equal to the cutoff value, while “high expression” tumors were those with scores above the value. To facilitate ROC curve analysis, the clinicopathologic features were dichotomized: survival status (death due to lung adenocarcinomas versus censored).

### Preparation of DC nanoparticles and encapsulation siRNA

DOTAP-cholesterol (DC) was purchased from Avanti Polar-lipids Inc. (Birmingham, AL, USA). HPLC-grade chloroform were obtained from Sigma Chemical Co. (St. Louis, MO, USA).The nanoparticles were prepared by an EmulsiFlex-B3 high-pressure homogenizer (HPH) (Avestin Inc., Ottawa, Canada) [24], [25]. The nanosomes were kept at room temperature for 1 h prior to overnight storage at 4°C.

CPSF4 siRNA and nonspecific siRNA were purchased from Shanghai GenePharma Co. (Shanghai China). The sequences of the human CPSF4-specific siRNA were 5'-CAU GCA CCC UCG AUU UGA ATT-3' (siRNA-1)and 5'-GGU CAC CUG UUA CAA GUG UTT -3' (siRNA-2); nonspecific siRNA, 5'-UUC UCC GAA CGU GUC ACG UTT-3'. The CPSF4 overexpression vectors pcDNA3.1-CPSF4 and the control vector pcDNA3.1 were designed and synthesized by Cyagen (Cyagen Biosciences Inc., United States). For delivery of CPSF4 siRNA or CPSF4 expressing vector into lung cancer cells, the siRNA or plasmid vector was encapsulated into DC nanosomes which had validated previously [26]-[28].

### Cell proliferation and colony formation assay

Cell proliferation was assessed using the MTT assay (Promega) according to the manufacturer’s protocol. Cell viability was determined after transfection with CPSF4 siRNA or CPSF4 expressing vectors. At 48 hours after treatment, cells (H1299 and A549) were harvested, counted, and single cells seeded (400 cells/well) in triplicate into 6-well plates. Cells were treated with CPSF4 expressing vectors or CPSF4 siRNA every 3 days and grown for 8-14 days [29]; colonies were stained with crystal violet for 10 minutes at room temperature. Colonies that contained more than 50 cells were counted.

### In vitro invasion assay

The *in vitro* invasion chamber assay was conducted with Millicell Cell Culture Insert (24-well PCF 8.0 µl, Millipore). Briefly, the inserts standing in 24-well cell culture plate were coated with 100 µl 1 mg/mL Matrigel Matrix (BD Falcon, USA) in serum-free RPMI-1640 medium and air-dried. 48 hours after CPSF4 siRNA treatment, cells (H1299 and A549) were harvested, counted, and 100 µl serum-free medium containing 5×10^4^ cells were added to the insert. The lower compartment contained 0.5 ml of RPMI-1640 medium containing 10% FBS. This follows incubation in 5% CO_2_ at 37% for 24 hours. Then we fixed the cells that penetrate the membrane into the lower side with formalin and these cells were stained by crystal violet. The invasiveness of the cells was determined by counting the penetrating cells onto the lower side of the filter through the pores under a microscope at x100 magnification. We assayed 3 times and randomly selected 3 fields were counted for each assay.

### Apoptosis and cell cycle analysis

For detection of apoptosis and cell cycle, cells (H1299 and A549) transfected with siRNA were analyzed by flow cytometry. At 72 h after transfection, cells (1×10^5^ cells/ml) were stained with 5 µl AnnexinV-FITC and 5 µl PI (propidium iodide) using an Annexin V-FITC/PI-staining kit (Becton Dickinson, CA, USA), placed on room temperature for 15 min in the dark, and then analyzed by flow cytometry (EPICS XL; Beckman Coulter). Apoptosis was calculated in terms of the FITC-positive in cells. Cell cycle analysis was performed using PI staining. The transfected cells were harvested with trypsinization, fixed in 70% cold ethanol for 30 minutes. After treatment with 100 mg/ml RNase (Sigma–Aldrich), the cells were stained with 50 mg/ml PI (Sigma Aldrich) in PBS. Flow cytometry (EPICS XL; Beckman Coulter) was performed immediately. Raw data were analyzed using Multicycle for Windows (Beckman Coulter).

### Statistical analysis

Student’s t-test was used to compare two independent groups of data. The median immunohistochemical staining score was used as the cutoff value to divide the patients into low and high CPSF4 expression groups. Chi-square tests were applied to analyze the relationship between CPSF4 expression and clinicopathologic status. Kaplan–Meier survivals were plotted and log-rank tests were performed. Univariate and multivariate analyses were done with the Cox proportional hazard regression model to determine associations between clinicopathologic variables and cancer-related mortality. A *P* value <0.05 was considered statistically significant in all cases.
